# Remote-Management of COPD: Evaluating the Implementation of Digital Innovation to Enable Routine Care (RECEIVER): the protocol for a feasibility and service adoption observational cohort study

**DOI:** 10.1136/bmjresp-2021-000905

**Published:** 2021-08-30

**Authors:** Anna Taylor, David J Lowe, Grace McDowell, Stephanie Lua, Shane Burns, Paul McGinness, Christopher M Carlin

**Affiliations:** 1 Respiratory Medicine, Queen Elizabeth University Hospital, Glasgow, UK; 2 Emergency Medicine, Queen Elizabeth University Hospital, Glasgow, UK; 3 Lenus Digital Health, StormID, Edinburgh, UK

**Keywords:** COPD exacerbations, emphysema, non invasive ventilation

## Abstract

**Introduction:**

Reductions in exacerbation and hospitalisations are the outcomes rated as most important by people with chronic obstructive pulmonary disease (COPD). Most COPD management is currently based on a reactive approach, and delays in recognising treatable opportunities underpin COPD care quality gaps. Innovations that empower COPD self-management, facilitate integrated clinical care and support delivery of evidence-based treatment interventions are urgently required.

**Methods and analysis:**

The Remote-Management of COPD: Evaluating the Implementation of Digital Innovation to Enable Routine Care trial is a prospective observational cohort hybrid implementation and effectiveness study that will explore the adoption of a digital service model for people with ‘high-risk’ COPD and evaluate the feasibility of this approach versus current standards of care. People with COPD, who have had recent severe exacerbation and/or COPD–obstructive sleep apnoea overlap or chronic hypercapnic respiratory failure requiring home non-invasive ventilation (NIV) or continuous positive airway pressure (CPAP), with internet access will be recruited into the study and enrolled into the digital service.

Study endpoints will examine participant utilisation, clinical service impact and clinical outcomes compared with historical and contemporary control patient data. The digital infrastructure will also provide a foundation to explore the feasibility of approaches to predict outcomes and exacerbation in people with COPD through machine learning analysis.

**Ethics and dissemination:**

Ethical approval for this clinical trial has been obtained from the West of Scotland Research Ethics Service. The trial will commence in September 2019 for a duration of 2 years. Results will be presented at local, national and international meetings, as well as submission for publication to peer-reviewed journals.

**Trial registration number:**

NCT04240353.

## Introduction

Chronic obstructive pulmonary disease (COPD) is a common, preventable and treatable disease, which is characterised by persistent respiratory symptoms and airflow limitation. The main risk factor for COPD is tobacco smoke but other environmental exposure may contribute. Respiratory symptoms include breathlessness, cough and/or sputum production. These symptoms are under-reported by patients.^1 2^ COPD may be punctuated by periods of acute worsening of these respiratory symptoms, often referred to as exacerbation, that can result in emergency department (ED) attendance or hospital admission. For many patients, COPD is associated with significant comorbidity, which increases its morbidity and mortality.

COPD is a major healthcare challenge, with worldwide rising prevalence. The Global Burden of Disease Study reported a prevalence of 251 million cases of COPD globally in 2016.^3^ It was projected to be the fourth leading cause of death worldwide by 2020.^4^ Reductions in exacerbation and hospitalisations are the outcomes rated as most important by patients with COPD.^5^ Effective delivery of evidence-based interventions for COPD—smoking cessation, influenza vaccination, pulmonary rehabilitation, personalised inhaled therapy, home oxygen therapy (where indicated) and home non-invasive ventilation (NIV, where indicated)—has been shown to reduce exacerbation and hospital admission.^6^ There are considerable barriers to uptake and delivery of these interventions.^7^ This care quality gap particularly affects outcomes from COPD exacerbation. Exacerbation is the main driver of healthcare costs (estimated annual National Health Service (NHS) cost of managing COPD is £1.9 billion.^8^ There is an urgent requirement for an innovation-based service redesign that can integrate care to deliver these evidence-based interventions and achieve reductions in COPD exacerbation and admissions.

Self-management also plays a key role in the treatment of COPD.^9^ Patients who can be successfully taught and supported with COPD self-management show a significant reduction in COPD admissions.^10 11^ The process of establishing a multidisciplinary community respiratory team in NHS Greater Glasgow & Clyde (NHS GG&C), which supports self-management in patients identified acutely as being high risk of hospital admission, has been associated with reduction in hospital admission rates.^12^

While interventions should be developed for patients with all severities of COPD, it is logical to target immediate efforts towards patients with ‘high-risk’ COPD, that is, those who are at most risk of exacerbation and hospital admissions. Established data indicate that patients with COPD who have had severe exacerbation (one requiring ED attendance or hospital admission) in the previous 12 months and/or have persisting hypercapnic respiratory failure requiring home NIV fall into this high-risk group.^6 13^ Interventions proven in this group can then be rolled out (if cost-effective) to the lower risk groups of patients with COPD.

### Digital service model

Pilot data from NHS GG&C have highlighted the potential for digital innovations to predict COPD outcomes and support treatment uptake. Using qualitative methods, Slevin *et al* highlighted patient acceptance to take an active role in self-management using digital health technology with the potential for healthcare professionals to provide meaningful preventative care.^14^ Web and smartphone-based apps have shown the capability to facilitate disease self-management and support uptake of interventions.^15 16^ Although patient-focused digital tools currently exist for COPD, there is a limited evidence base for their use, with evaluations mainly performed in isolation and not integrated with established clinical services or statutory electronic health records (EHRs).

Most COPD management is currently based on a reactive approach, and delays in recognising treatable opportunities underpin COPD care quality gaps. For example, patients with COPD exacerbation typically have symptom deterioration for 2 days before seeking assistance, and then a potential delay of 2–5 days in accessing clinical care.^1^ Several studies have indicated the ability of regularly recorded patient-reported outcomes (PROs) and home NIV parameters to predict outcomes, including exacerbation and treatment success/failure in patients with COPD.^17 18^ Changes in activity measured by wearable devices have been shown to predict outcomes after COPD exacerbation.^19^ Currently, symptom diaries and other PRO questionnaires, activity, exercise and NIV data are obtained in routine practice in NHS GG&C. However, patient and clinician engagement is not consistent, data are not acquired systematically and are not often visible or actionable at key time points of patient–clinician interaction. These shortfalls, and the arising care quality gaps, could potentially be addressed by digitising this routine clinical care, improving the patient–clinician interface for data entry, and collating the acquired data.

Patient–clinician communication for COPD management, including supporting self-management, is currently dependent on face-to-face scheduled consultations, answer phone/email asynchronous messages from patient to clinician, and unstructured advocacy triggered or initiated communication from clinician to patient. These present several inefficiencies and risks. They could be overcome by digitalising the patient–clinician messaging system to support scheduling, remote management and support COPD self-management.

Home NIV successfully improves admission-free survival in patients who have persisting hypercapnic respiratory failure following life-threatening COPD exacerbation, with a number needed to treat of seven patients.^20^ NHS GG&C has successfully matched the outcomes from the HOT-HMV randomised clinical trial in our service adoption pilot delivering home NIV to eligible patients with COPD, using routinely available digital technologies (adaptive ‘auto-NIV’ modes, two-way remote monitoring via ResMed AirView platform).^21 22^ The challenge is to extend the evidence for this approach and obtain a service adoption playbook to enable this to be adapted and delivered at scale, by other clinical teams, within COPD integrated care.

Machine learning analysis and modelling based on available data shows significant promise in COPD predictive management. Data available in patient’s EHR at triage assessment can robustly predict outcome (admission, length of stay) from severe COPD exacerbation.^23 24^ The addition of physiology measurements to EHR data improves machine learning predictive model performance in other clinical conditions.^25^ Further evaluation of these analytics and predictive modelling, in a comprehensive dataset including PROs, physiology data and clinical events, is a logical step to determine their potential role in real-time or near real-time clinical use.

### Objectives

Innovations which can empower patient self-management, facilitate integrated clinical care and support delivery of evidence-based treatment interventions are urgently required. In the Remote-Management of COPD: Evaluating the Implementation of Digital Innovation to Enable Routine Care (RECEIVER) trial, we propose to explore the implementation of a platform which digitalises these as additional—potentially assistive—components alongside routine clinical care. In our endpoints, we will determine participant utilisation, clinical service impact and clinical outcomes, to evaluate the feasibility of this approach versus current standards of care. A digital infrastructure for this support of routine clinical care would also provide a foundation to explore the feasibility of approaches to predict outcomes and exacerbation in patients with COPD, to be tested in future prospective clinical and regulatory trials.

Our aims are to establish a digitalised service model for ‘high-risk’ patients with COPD which will:

- Integrate current routine clinical care within a digitally enabled remote management service infrastructure.
- Enable delivery of remote management of COPD at scale within the NHS and other healthcare systems.
- Capture relevant routinely acquired PROs, continuous physiology data and clinical event/episode data in a patient and clinician co-designed interface which enables engagement.
- Facilitate evolution from a reactive to a proactive and preventative COPD service model.

### Digital service components

**Figure 1:**
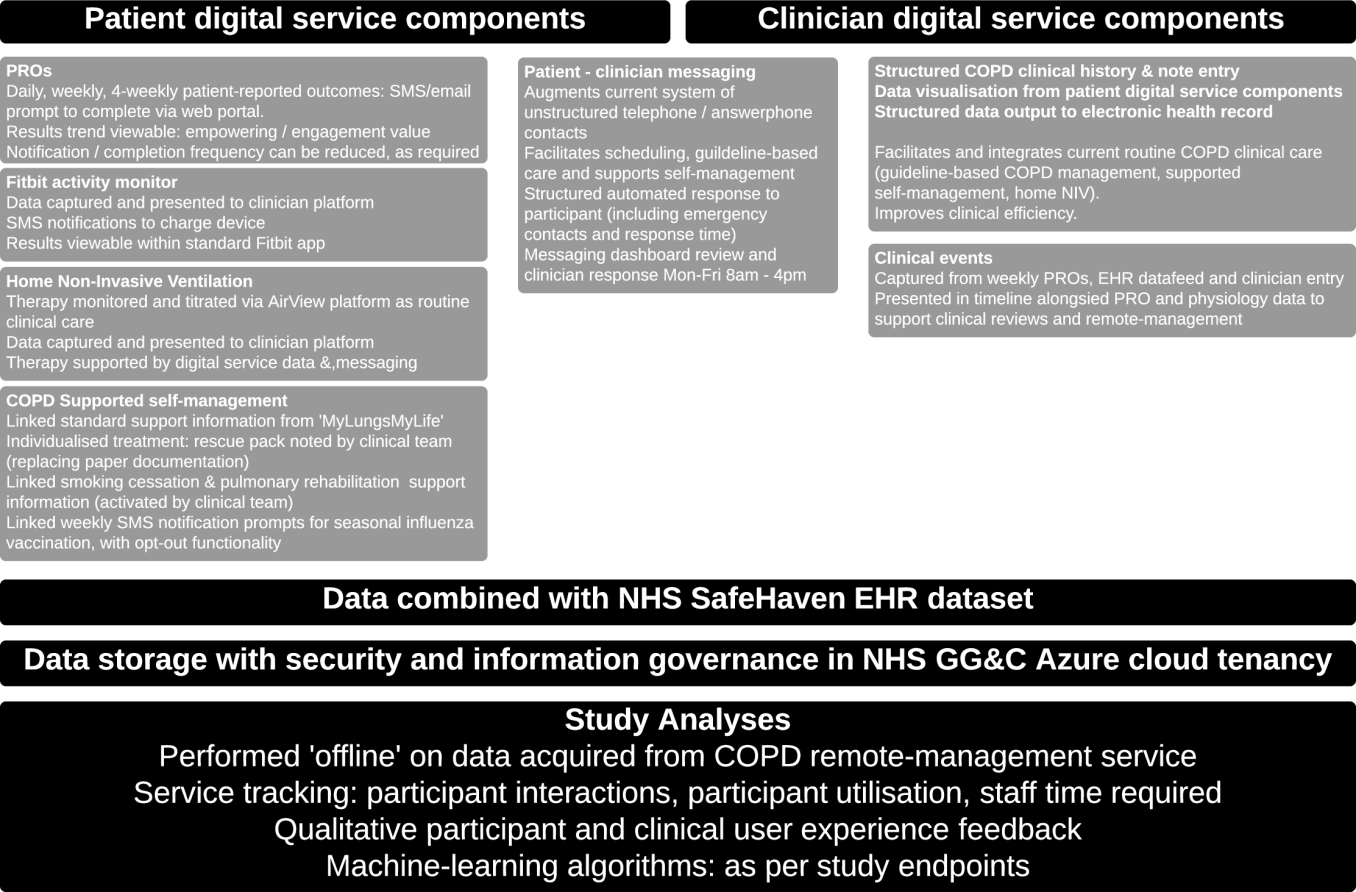
RECEIVER Digital Service ComponentsCOPD, chronic obstructive pulmonary disease; EHR, electronic health record; NHS GG&C, National Health Service Greater Glasgow & Clyde; NIV, non-invasive ventilation; PROs, patient-reported outcomes; SMS, Short Message Service.

The digital service model will sit alongside routine clinical care, aiming to assist and enhance current patient management. Key components of proposed digital service model for COPD are noted and summarised in figure 1. Tabulated information in online supplemental material 2 outlines how the RECEIVER trial components would be used in addition to current routine clinical care.

10.1136/bmjresp-2021-000905.supp2 Supplementary data

The COPD digital service components being used in the RECEIVER trial are:

- Patient-facing web portal: this has been co-designed with patients and captures PROs and provides access to standard COPD self-management content. It also includes a messaging facility which can be patient or clinician initiated. Examples of visuals available on supporting website (https://support.nhscopd.scot).
- Patient wearable device: Fitbit Charge 3 device (CE marked).
- Remotely monitored home NIV: provision of this is our current standard of care for patients with hypercapnic respiratory failure, using AirView NIV remote management platform (ResMed). We have integrated our COPD digital service platform with the AirView platform, so that it acquires the NIV data unmodified for review where available. NIV therapy management will continue to be conducted through the AirView platform: the RECEIVER trial COPD platform is not used to modify or enhance patient NIV therapy.
- User-designed clinical dashboard: presents integrated data with an aim of facilitating improvement in provision of guideline-based COPD care, and supporting COPD self-management.
- Patient–clinician asynchronous messaging: to support routine clinical care.
- NHS GG&C Azure cloud-based digital architecture: this provides and integrates the above services with existing NHS GG&C electronic healthcare systems. Diagram of digital architecture included in online supplemental material 3.

10.1136/bmjresp-2021-000905.supp3 Supplementary data

## Study methods and analysis

### Study design

This study is a prospective observational cohort hybrid implementation and effectiveness study.^26^ It will be performed according to the UK Policy Framework for Health and Social Care Research (2020).^27^ The clinical intervention component is regarded as a phase IV adoption study. Data visualisation to facilitate guideline-based care, supported self-management and home NIV are evidence-based COPD interventions.

In this study, we will evaluate the adoption of digitally integrated remote management service innovations to support routine clinical care. Implementation of these will be evaluated and the digital innovations will be iterated to optimise them, based on user experience accrued during the study. We will acquire a consented dataset for trial endpoint analysis including exploratory machine learning-based predictive modelling. The machine learning analysis will also allow us to prioritise data inputs for follow-up study.

Secondary outcomes for the RECEIVER cohort will be compared with those from matched cohorts of patients acquired from the NHS GG&C SafeHaven dataset. Ethics approval is in place to access this large database of de-identified data, to establish a control cohort from a historical time series and a RECEIVER trial concurrent time series. These cohorts will be matched for demographics, COPD severity and hospital admission frequency with the RECEIVER cohort.

We will conduct a substudy of additional baseline and follow-up physiological measurements (oscillometry, parasternal electromyography (EMG), home pollution monitoring) in patients in whom it is feasible to obtain these during their hospital admission and/or hospital attendance alongside their routine clinical care.

Additionally, we will conduct a substudy of clinical users, with platform tracking analytics to measure clinician time spent on the platform, and user experience data. Clinical team members involved in the delivery of patient support using the digital service will be eligible for inclusion and appropriate consent will be sought. The qualitative components will be acquired through semistructured group or 1:1 interviews conducted by trained members of the research team. Thematic analysis will be performed. Anonymised transcripts will be coded, and themes generated from the codes. Themes will be discussed within the research team to reach agreement and add rigour.

**Figure 2:**
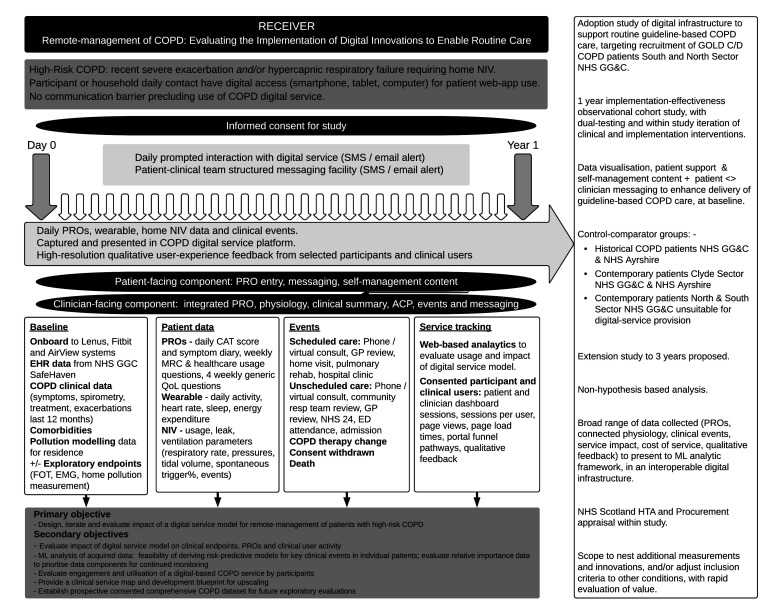
RECEIVER Study DesignACP, anticipatory care plan; CAT, chronic obstructive pulmonary disease assessment tool; COPD, chronic obstructive pulmonary disease; ED, emergency department; EHR, electronic health record; EMG, electromyography; FOT, forced oscillation technique; GOLD, Global Initiative for Chronic Obstructive Lung Disease; GP, general practitioner; HTA, Health Technology Assessment; ML, machine learning; MRC, Medical Research Council dyspnea scales; NHS, National Health Service; NHS GG&C, National Health Service Greater Glasgow & Clyde; NIV, non-invasive ventilation; PROs, patient-reported outcomes; QoL, quality of life; SMS, Short Message Service.

An overview of the study design is provided in figure 2.

### Study population

People with high-risk COPD attending secondary care in the North and South Sectors of NHS GG&C will be screened for inclusion in this study.

Inclusion criteria:

- Confirmed diagnosis of COPD, established prescreening or at screening, defined as per Global Initiative for Chronic Obstructive Lung Disease (GOLD) 2020 guidelines.^6^
- Home NIV cohort: hypercapnic respiratory failure and/or sleep disordered breathing, meeting established criteria for provision of home NIV.
- Exacerbation cohort: presentation within last 12 months with severe or life-threatening exacerbation of COPD, defined as per GOLD 2020 guidelines.^6^
- Participant or close contact who has daily access to smartphone, tablet or home computer with internet access.
- Informed consent.
- >18 years of age.

Exclusion criteria:

- Inability to comprehend informed consent.
- Communication barrier precluding use of COPD digital service.

### Sample size

Previous NHS GG&C studies,^10 21^ combined with internal audit data (showing that 80% of patients have direct or daily smartphone, tablet or web access), indicate that recruitment of 400 participants across a 12-month period is feasible, and likely to provide adequate data to conduct meaningful primary and secondary endpoint analyses.

An improvement in admission-free survival, with reduction in admissions by one per patient per year is projected, if support for provision of evidence-based COPD care is achieved by the service model. As the feasibility of an end-to-end digitalised service model for COPD has not yet been tested, and the service components will be adapted during the study, sample size power calculations and randomisation are not yet appropriate.

### Screening and eligibility assessment

Participants will be identified from ED attendance, hospital admission or COPD multidisciplinary team referral. It is anticipated that the majority of participants will be identified and recruited during their index acute admission.

Review of focused medical history and lung function test results will be undertaken as part of screening evaluation to ensure all inclusion and no exclusion criteria are met.

### Informed consent

We will undertake informed consent with timing which is individualised to be least burdensome and most efficient for participants. Written informed consent will be obtained by participant’s dated signature and dated signature of the person who presented and obtained the informed consent. The person who obtained the consent will be suitably qualified and experienced and have been given authority to do so by the principal investigator (PI).

In addition to consent for the clinical trial, General Data Protection Regulation (GDPR) consent for data sharing, data storage and tracking of web app activity is required, and this is sought within the COPD patient portal when participants complete initial sign-up to the service.

### Withdrawal of subjects

Participants have the right to withdraw from the trial at any point, for any reason, without prejudice to future care. This is clearly stated at time of consent. The investigator can also withdraw participants from the study intervention in the event of protocol violations or if it was felt that involvement in the service was adding unnecessary burden or distress (eg, if participant was approaching end of life). Participant withdrawals will be included within the study analyses and reporting.

Participants may opt to limit the number of interactions—for example, daily questionnaires, that they undergo, if these are burdensome. They may also request ‘conventional’ scheduled face-to-face clinical review rather than remote managed review at any time point. This will be accommodated as outpatient or inpatient attendances, within routine clinical timescales as judged appropriate by the clinical team, in line with current service provision.

### Participant identification

Study numbers will be assigned sequentially as each subject enters the study to ensure that study participant data are de-identified. Corresponding information will be recorded on the Case Report Form (CRF) by the investigator.

### Source data

Source documents will include hospital medical records, clinical charts, laboratory and pharmacy records, radiographs and correspondence. The patient web portal and clinician dashboard will be the source data for clinical summary data, PROs, wearable and NIV physiology, patient–clinician messaging, clinical documentation and exacerbation/admission recording in participants. The clinician dashboard will also output this source data as structured report to NHS GG&C clinical portal (patient EHR).

### Study data

Electronic data and COPD service digital architecture will be held within NHS GG&C e-health systems, with industry standard security and identity assurance processes. The core components of digital service will be on NHS GG&C Azure cloud tenancy, further ensuring security and identity assurance, and avoid need for data de-identification prior to machine learning analysis.

Data access will be password protected and accessible only by study investigators, with data management as per NHS GG&C and NHS Scotland data protection policies. Platform analytics will track user interactions and user data changes, to provide an audit trail for data integrity.

### Subsequent assessments

Participants will be recruited into the digitalised service model over a 12-month period. Observation of secondary endpoints will be continued over the 12-month period following recruitment. Participants who are recruited will subsequently continue within the digital service model beyond the trial period, either until ongoing procurement of the service infrastructure is established, or transition to alternative equivalent service model is completed.

### Definition of end of trial

The end of trial will be when the last participant has completed follow-up, 12 months after recruitment.

### Patient and public involvement

Patients and members of the public were involved throughout the planning and conduct of this research. The patient-facing web portal which forms the basis of the study was co-designed with patients with COPD and their carers. Semistructured interviews were conducted between patients and the development team to understand patient experience of their condition, with feedback and adaptation of the application at each development stage. Subsequent development of research questions and study design was informed by insights gained through these interactions.

### Study limitations

We recognise that there is an inherent risk of bias and limitations to using this type of study design with regard to the conclusions that can be drawn from the study outcomes. With this acknowledgement, our study seeks to explore the feasibility of using this digital service model within a real-world setting as opposed to providing evidence of definitive proof or utility. If feasibility is established, the format and experience gained from conducting this initial research will form the basis for future randomised studies.

## Data collection

### Data collection by research team

#### EHR data

The following data will be obtained from EHRs held within NHS GG&C SafeHaven: demographic, coded diagnosis list, Charlson Comorbidity Index, Scottish Index of Multiple Deprivation quintile, medication history, laboratory results, lung function results, ED attendance, hospital admission, pulmonary rehabilitation attendance data from 1 January 2010 onwards. These data will be presented for evaluation in a study machine learning analysis platform.

#### Baseline clinical data

The following data will be collated when the participant is enrolled and added to the clinical details form visible on the clinician dashboard: participant age, sex, smoking status, vaccination status, comorbidities, COPD exacerbation history, COPD medications, lung function results, key laboratory results including maximal eosinophil count and modelled home pollution exposure (QCumber platform; NO2, O3, PM2.5, black carbon).

Participants who have not had contemporary spirometry (standard lung function test) will have this repeated at the time of recruitment—this would be routine clinical care.

#### Follow-up clinical data

Smoking cessation, vaccination status, pulmonary rehabilitation, COPD comorbidity, COPD exacerbation history, COPD medications and other treatments, inhaler technique and narrative history will be updated at clinical encounters, as relevant.

A data feed from NHS GG&C Trakcare platform to RECEIVER Study COPD platform will present information on hospital attendance and admissions.

Clinical episodes are also captured in the weekly PRO questionnaires, and manually inputted by the clinical team, when these are noted.

Accuracy of coding of clinical summary data and clinical episodes will be reviewed in a proportion of recruited participants, by a respiratory physician working independent of this study.

#### Physiology substudy

A subset of participants (where time and mode of presentation allow) will be offered recruitment to the exploratory physiology substudy and have additional physiology measurements taken. These will comprise of oscillometry, parasternal EMG and home air pollution pack monitoring. Measurements from these may predict/associate with stability of COPD and clinical endpoints, and follow-up measurements may be taken at 3, 6, 9 and 12 months if feasible during study, alongside routine clinical care. The principal purpose of conducting these in this study is to report on feasibility within routine COPD assessment.

### Data from PROs

Participants (or their family or carer) will complete PRO questionnaires in the patient web portal. Daily SMS and/or email notifications are provided as a prompt to complete these. There is support information in the associated website to assist participants with any difficulties with the patient portal. Additional support will be provided by the clinical users if required.

Participants will complete symptom diary and COPD assessment tool (CAT) questionnaire daily. Medical Research Council dyspnea score (MRC) and healthcare episode questionnaires will be completed weekly. EQ-5D-5L generic quality of life questionnaire will be completed every 4 weeks. These questionnaires have been integrated to improve question flow and simplify the user experience. Questionnaire flow content is included in the online supplemental material 1.

10.1136/bmjresp-2021-000905.supp1 Supplementary data

PRO data are presented unmodified, in a ‘user-friendly’ format (co-designed with clinical users), in the clinician dashboard. These unmodified data will inform and enhance clinical encounters and patient–clinician communication.

Pilot user experience research shows that the daily PRO questions can be completed in ~70 s, with weekly questions taking an additional 90 s, and monthly generic quality of life questions taking an additional 5 min.

Specific permission for use of CAT questionnaire within this e-health system has been obtained. The use of other PROs is covered by available open licensing.

### Wearable physiology measurement

Participants will be provided with a Fitbit Charge 3 wristband wearable device, to monitor activity, sleep, heart rate and energy expenditure variables.

Wearable physiology results and device support instructions will be available to participants in the Fitbit app.

Wearable physiology results are presented unmodified in the clinical dashboard, to inform and enhance clinical encounters and patient–clinician communication.

### Remote-monitored home NIV

Participants with severe COPD who have standard indications (persistent hypercapnia with PCO_2_ >7 kPa and/or recurrent episodes of acute hypercapnic respiratory failure and/or COPD–obstructive sleep apnoea overlap and/or COPD with significant nocturnal hypoventilation) are commenced on home NIV in NHS GG&C using Resmed Lumis 150 ST-A device as routine clinical care. Participants with significant obstructive sleep apnoea syndrome may be alternatively commenced or transitioned to auto-titrating continuous positive airway pressure (CPAP) using an AirSense-10 device. NIV initiation and optimisation will be conducted as per standard NHS GG&C clinical protocol. NIV data capture and device adjustment will be via ResMed AirView platform, again as routine clinical care.

AirView data will be acquired via an open application programming interface (API) to the COPD platform and presented in the clinical dashboard, to improve data visualisation for NIV clinical management alongside PROs and wearable data, and to inform and enhance other clinical encounters and patient–clinician communication.

### Patient portal support materials, care plans and clinical decision support

The patient web portal will contain linked content to NHS GG&C smoking cessation, vaccination, inhaler therapy, pulmonary rehabilitation, breathing control and ‘my lungs, my life’ COPD support literature, all to aid self-management.

The patient resource within the patient portal will contain a structured exacerbation self-management care plan. This is a digitalised version of the paper structured self-management plan without any change in format or content. Participants will be empowered to activate their own self-management plan or supported/directed to activate this with patient–clinician communication, as standard routine clinical care: the data access, visualisation and patient–clinician messaging facilities in this study will however enhance this.

### Machine learning analysis and predictive modelling

These analyses will be conducted post hoc, using the clinical data obtained from the RECEIVER trial COPD platforms, alongside EHR data in historical and contemporary control patients from NHS GG&C SafeHaven. The anticipation is that following analysis of the study data, we will be able to judge the feasibility and accuracy of using predictive model outputs at a service or individual patient level. This may lead to the subsequent development and evaluation of artificial intelligence(AI)-based clinical decision support tools. Relevant clinical investigation/medical device trial(s) of these would be proposed and designed. The data from the RECEIVER trial would not be used directly to support clinical validation of any subsequently developed medical device.

### Service model iterations

The components of the RECEIVER service model have been subject to pilot evaluation and pretrial patient and clinical user experience research. Service refinement based on the clinical user substudy experience research will continue during pretrial preparation and approval period. Iterations of the digital service content (eg, rationalisation of patient outcomes, modification or addition of self-management support materials) based on the adoption experience will be considered by the project steering group at three monthly intervals during the study. Where this iteration would result in a change in the clinical protocol, this will be submitted as an amendment for consideration and regulatory approval before any change is implemented.

## Study outcome measures

Primary and secondary endpoints for the RECEIVER Study are shown in table 1.

**Table 1:** RECEIVER primary and secondary endpoints

Primary endpoint	Proportion of enrolled high-risk participants with COPD who use remote management in a digital service model
Secondary endpoints	Clinical outcomes, comparing impact of digitally enabled remote management versus historical and contemporary SafeHaven cohorts: Clinical events: COPD exacerbation; unscheduled care contact (digital platform, COPD team visit, primary care, emergency department, hospital admission); mortality—COPD and non-COPD related. Hospital occupied bed days preceding and subsequent 12 months (adjusted time interval if survival <12 months). Treatment uptake (where indicated): smoking cessation; pulmonary rehabilitation; vaccination; supported self-management; home oxygen; home NIV. NIV group: NIV usage, symptom change, NIV therapy parameters, blood gases during routine clinical care. Supported self-management: utilisation of self-management information (page views), number of exacerbation managed at home versus in hospital, number of rescue packs used in 12 months (captured through weekly PROs), utilisation of messaging (number of message threads), sputum microbiology (where available during routine clinical care), impact of patient activation measures (where measured during routine care). Impact of demographics, physiology and patient activation measures (where measured during routine care)—deprivation category of area of residence, age and sex, number of previous admissions, smoking status, participation in pulmonary rehabilitation in previous 2 years; lung function measurements, modelled home air pollution exposure; EMG, oscillometry and home air pollution monitoring in subset of participants where this is carried out—on outcomes, clinical events and treatment uptake. Clinical service outcomes for digital service model, remote-managed home NIV and supported self-management: Remote-managed home NIV: number, nature and complexity of NIV therapy reviews and interventions to provide. Supported self-management: number, nature and complexity of reviews and interventions to provide. Digitalised service model: user and developer time/cost required for development and modification of clinical dashboard; qualitative analysis (clinical user satisfaction and reflection on efficiency or additional workload); quantitative analysis (clinical dashboard utilisation tracking). Patient portal: user and developer time/cost required for development and modification of clinical dashboard; qualitative analysis (user satisfaction) and quantitative analysis (uptake, engagement with app and wearable, successful use of digital service vs bypass to conventional healthcare contacts). Machine learning-supported exploratory analyses of associations and relative predictive importance of electronic health record, PROs, wearable physiology and NIV parameters: Associations between changes in PROs (MRC, CAT, symptom diary, and EQ-5D-5L questionnaires) with routine clinical care interventions, COPD exacerbation and other clinical events. Associations between changes in wearable monitoring parameters (activity, sleep, heart rate variability, energy expenditure, respiratory rate) with routine clinical care interventions, COPD exacerbation and other clinical events. Associations between changes in NIV-monitored parameters (usage, leak, airway pressures, respiratory rate, tidal volume, minute ventilation, inspiratory/expiratory ratio and detected respiratory events) with routine clinical care treatment interventions, COPD exacerbation and other clinical events. Associations between changes in clinical endpoints and relative importance plots of all remote management-acquired data (including EMG, oscillometry and home pollution monitoring exploratory endpoints in subgroup these measured on) to determine contribution of these to outcome prediction, and therefore value of these for future prospective study. Patient-centred outcomes: Health-related quality of life (EQ-5D-5L) at baseline and monthly during study. Qualitative user research (planned subset of participants, convenience sample) with semistructured user experience interviews. Impact of patient activation (where this is measured at baseline and/or follow-up during routine clinical care) on enrolment and use of digital service model. Healthcare cost analyses: Development and installation costs for digitalised service model for remote management of COPD. Recurring costs (clinical staffing, digital platform hosting, digital platform scheduled update and maintenance) for digital service model for remote management of COPD. Projected direct and indirect cost-savings (admission and unscheduled care reduction, travel, carer burden impact, clinical efficiency) of high-risk COPD with digitally enabled remote management, compared with previous service model.

CAT, Chronic obstructive pulmonary disease assessment tool; COPD, chronic obstructive pulmonary disease; EMG, Electromyography; MRC, Medical Research Council dyspnea scale; NIV, non-invasive ventilation; PROs, patient-reported outcomes; RECEIVER, Remote-Management of COPD: Evaluating the Implementation of Digital Innovation to Enable Routine Care.

### Primary endpoint measures

The proportion of participants who use the digital service model will be determined from screening versus recruitment log, and with participant usage (PRO completion, wearable usage, home NIV usage and digital service contacts vs conventional healthcare contacts), monitored using consented platform tracking analytics. A target of submission of at least one PRO set per participant per week will be used to ascertain utilisation measures.

### Secondary endpoint measures

Clinical events, hospital occupied bed days, treatment uptake, NIV usage and therapy, incidences of supported self-management and the impact of demographics on these will be captured within the digital service platform with data from weekly SafeHaven export, Trakcare real-time episode data feed and platform tracking analytics.

The process evaluation metrics for the digital service model will be captured by platform tracking analytics, project delivery process documentation and supplementary user experience research conducted as part of the digital service project delivery.

Machine learning-supported analysis of study dataset will be conducted post hoc.

Patient-centred outcomes will be captured within RECEIVER patient portal.

Qualitative user experience research will be undertaken with a subgroup of participants to inform implementation strategy and will be presented as a descriptive summary. A sample of convenience will be taken from within the RECEIVER cohort. Semistructured interviews will be conducted, and thematic analysis will be performed. Interview transcripts will be coded, and the codes used to generate themes. Discussion of the themes within the research team will be conducted to ensure agreement and add rigour.

Cost analysis will be based on audited accounts for the RECEIVER innovation project delivery, combined with standard NHS tariffs, NHS Scotland medical/agenda for change salary scales and indirect costs from standard burden of COPD cost projections.

## Data management

The study data file will be held in a locked cabinet in the Department of Respiratory Medicine, Queen Elizabeth University Hospital, Glasgow, with an electronic copy securely stored on the EDGE Clinical Research Management System. All electronic data and COPD service digital architecture (including machine learning algorithms) will be held within NHS GG&C e-health systems, with industry standard security and identity assurance processes. The core components of the digital service will be held within NHS GG&C Azure cloud tenancy, further ensuring security and identity assurance, and avoiding the need for data de-identification prior to machine learning analysis.

Data access will be password protected and accessible only by study investigators, with data management as per NHS GG&C and NHS Scotland data protection policies. Platform analytics will track user interactions and data changes, to provide an audit trail for data integrity.

At study completion, the comprehensive study dataset will be submitted for inclusion in NHS GG&C SafeHaven. This will allow (with appropriate SafeHaven standard operating proceedures (SOP) and Local Privacy and Advisory Committee (LPAC) application approvals) subsequent de-identified review of all study outcomes, reanalysis of the dataset, and contribution of the dataset to future COPD data linkage and other research work within NHS Scotland.

## Statistics and data analysis

Secondary outcomes in the RECEIVER observational cohort will be compared with matched retrospective data from de-identified linked datasets of historical control and contemporary control high-risk patients with COPD. These will comprise of patients identified from coding and admission data as having had severe exacerbation of COPD between 1 January 2010 and 30 April 2019 (historical cohort) and between 1 July 2019 and 30 June 2020, excluding participants enrolled in the RECEIVER Study (contemporary control). The historical control dataset will contribute to machine learning models development. Secondary outcome analysis will be separately compared between the RECEIVER cohort, historical cohort and contemporary control cohort. This component of the study is also separately registered with dataset access approval from NHS GG&C SafeHaven LPAC.

The clinical endpoint data will be reported as a proportion and 95% CI for that outcome, calculated by Kaplan-Meier method. Demographic data will be presented as mean and SDs, or medians and IQRs as appropriate. Correlation, t-test, analysis of variance and effect size analyses will be used as appropriate for secondary endpoint analysis, comparing results of clinical endpoint and patient-centred outcomes with NIV therapy and supported self-management parameters.

Machine learning ensemble methods will be used to develop binary classification problems for patient risk. Precision, recall, accuracy and C-statistics of the receiver operating characteristic curve will be used to evaluate model performance.

## RECEIVER trial qualitative substudy: protocol addition and amendment September 2020

Following the outbreak of the COVID-19 pandemic, the digital service model established in the RECEIVER trial was adopted for routine clinical care in NHS GG&C. This was to mitigate for the anticipated COVID-19 impacts on routine COPD care, with concern about increased winter admission risks and continued requirement to maintain social distancing with vulnerable/shielding patients. Process for invitation and remote enrolment in the COPD digital service, via support website, has been established. People with COPD are enrolled following clinician referral or via invitations sent by Short Message Service (SMS) and letters to known patient cohorts.

Patient utilisation, clinical and service outcomes in this scale-up cohort will be evaluated in parallel with the data from the RECEIVER trial. These analyses will be conducted on de-identified data derived from NHS GG&C SafeHaven. There is separate ethics approval and protocol for these analyses.

It is relevant to gather user experience of the remote invitation and enrolment process to the digital service model and determine whether there are different experiences based on the recruitment source (via clinical trial, via invitation and website registration, via clinician referral). A sample of convenience of people who have enrolled in the COPD digital service via this scale-up service model will be established by the clinical team. They will be approached by the clinical team via the digital service messaging interface. People expressing an interest in undertaking semistructured interviews about their service experience will be sent a RECEIVER substudy patient information sheet and consent form. These people would then be contacted by one of the study investigators with the consent form discussed and completed by telephone, avoiding COVID-19 context risk of face-to-face contact for vulnerable individuals. The consent form with investigator signature would be mailed to the participant. Semistructured interviews would then be scheduled and conducted by telephone or video call, using NHS GG&C Attend Anywhere platform.

## Assessment of safety

The ‘RECEIVER’ digital service model is supporting rather than varying routine clinical care in people with COPD. Adverse device events and serious adverse events (ADEs and SAEs) will be captured within patient portal (eg, PRO question: have you had any hospital admissions?) and within COPD platform (by Trakcare-derived clinical events) and provided as a summary report.

Where an SAE requires recording, full details including the nature of the event, start and stop dates, severity, relationship to research product and/or trial procedures, and the outcome of the event will be recorded in the participant’s medical notes and CRFs. These events will be monitored and followed up until satisfactory resolution and stabilisation.

Each SAE will be assessed to determine if related to the research procedures and expectedness where the event is related by the following definitions:

- Related: that is, it resulted from administration of study medicines or any of the research procedures.
- Expectedness of SAEs: is against the research procedure events listed in study protocol as an expected occurrence.

### Expected adverse events

In general, there is little additional risk to participants taking part in the study. However, the study aims to digitalise existing reporting pathways and therefore the potential exists for failures to occur within the software used. Expected adverse events related to use of RECEIVER platform are as follows:

- Data connectivity issues: there may be issues with transfer of data from the participant to RECEIVER platform. Where these data relate to symptom diaries or Fitbit wearable data, there would be no impact on clinical care should the loss of data be temporary. However, in the event the data were permanently lost, this may impact on patient care.
- Messaging: there is a potential for messages sent from the participant to study staff (and vice versa) via the RECEIVER platform to be missed. There is also the potential for these messages to be confusing to the participant.
- Identity: there is the potential for data for one participant to be allocated incorrectly to another participant due to errors within the RECEIVER platform software.

Adverse effects unrelated to the use of the RECEIVER platform are not considered reportable to the sponsor.

Exacerbation of COPD resulting in hospitalisation and potentially death is expected within the study population. In addition, people with COPD may have other comorbid conditions. Events related to these conditions that meet the criteria of an SAE would also be considered expected. SAEs related to the participants’ underlying medical condition(s) that are not causally related to the RECEIVER platform are not reportable to the sponsor.

### Safety reporting to sponsor

The following events are reportable to the sponsor:

- Any SAE that is causally related to the use of RECEIVER platform (serious adverse device effect—SADE) regardless of expectedness.
- Any SAE that is related to the individual’s participation within the trial that is both related and unexpected but is not related to the use of the RECEIVER platform (related and unexpected SAEs—RUSAEs).

Study-related unexpected SAE must be reported to the Pharmacovigilance (PV) office immediately (within 24 hours). The SAE form should be completed and signed by appropriately delegated staff. If necessary, a verbal report can be given by contacting the PV office. This must be followed up as soon as possible with a signed written (or electronic) report. If all of the required information is not available at the time of initial reporting, the PI (or designee) must ensure that any missing information is forwarded to the PV office as soon as this becomes available. The report should indicate that this information is follow-up information for a previously reported event.

The PV office will report all RUSAEs and unexpected SADEs (USADEs) to the ethics committee within 15 days of the PV office becoming aware of the event, via the ‘report of SAE form’ for non-Clinical Trial of an Investigational Medicinal Products (non-CTIMPs) published on the Health Research Authority website.

RUSAEs and USADEs will also be considered individually by project steering group. Where appropriate, modifications (eg, additional patient alert notifications, additional clinical decision support notifications) to the RECEIVER platform will be made, in discussion with ethics committee and supported by protocol amendment.

Annual progress/safety report will be provided by the PI to the Research Ethics Committee(REC) and Research and Development (R&D) department.

### Protocol amendments

Any change in the study protocol will require an amendment. Any proposed protocol amendments will be initiated by the PI following discussion with the project steering group. Any required amendments forms will be submitted to the regulatory authority, ethics committee and sponsor. The PI and the project steering group will liaise with study sponsor to determine whether an amendment is non-substantial or substantial. All amended versions of the protocol will be signed by PI and sponsor representative. Before the amended protocol can be implemented, favourable opinion/approval must be sought from the original reviewing REC and R&D office(s).

## Device regulations

The COPD digital service patient and clinician applications are certified as class I under European Union Medical Device Regulations. The supplier (StormID) has a quality management system that is compliant with the requirements of International Standard BS EN ISO 13485:2016.

## Ethics and dissemination

This study will be carried out in accordance with the World Medical Association Declaration of Helsinki (1964) and its revisions (Tokyo (1975), Venice (1983), Hong Kong (1989), South Africa (1996) and Edinburgh (2000)).

Ethical approval for this clinical trial has been obtained from the West of Scotland Research Ethics Service. Participants will only be allowed to enter the study once they have provided written informed consent. This PI will be responsible for updating the ethics committee of any new information related to the study.

Key results will be presented at local, national and international meetings, including those with patient representation. All data that are obtained will be submitted for publication to peer-reviewed journals. Principal and co-investigators will have access to all data analyses conducted by the project commercial partners (StormID), with investigators having full academic independence for publication of results.
