# Supplementary material for: Remote-Management of COPD: Evaluating the Implementation of Digital Innovation to Enable Routine Care (RECEIVER): the protocol for a feasibility and service adoption observational cohort study
Source: BMJ Open Respir Res. 2021 Aug 30;8(1):e000905. doi: 10.1136/bmjresp-2021-000905 (PMC8407208; doi:10.1136/bmjresp-2021-000905)
Supplement: Supplementary data [file bmjresp-2021-000905supp002.pdf]

BMJ Publishing Group Limited (BMJ) disclaims all liability and responsibility arising from any reliance placed on this supplemental material which has been supplied by the author(s)

Supplementary material 2: RECEIVER Trial – Digitalising Routine COPD care

The components of the RECEIVER trial are a digitalisation of routine clinical care. These components, including integrated care and supported self-management are as recommended in international consensus guidelines for COPD management: Global Obstructive Lung Disease (GOLD) guideline 2019.

The table below summarises the routine clinical care currently available in NHS GGC and is shown alongside the trial schedule / study components that the digital service will add. These digital components are designed to assist and enhance current patient management but are not replacing current routine clinical care.

	Routine clinical care	RECEIVER trial
Symptom diary	CAT, MRC, symptom diary and generic-QOL questionnaires completed on paper at home and/or at COPD clinical reviews. Clinician aggregates and summarises data.	CAT, MRC, symptom diary and generic-QOL questionnaires completed by patient in webform.<br>Daily reminder text/email alert to complete.<br>Data presented unmodified to clinician in COPD dashboard.
Activity – exercise – sleep quantification	Narrative history +/- exercise and sleep diary completed on paper. Summary of patient Fitbit or other wearable device data reviewed when available. Actigraphy carried out by clinical physiology team and results collated in pdf report.	Patient provided RECEIVER study Fitbit.<br>Fitbit data acquired via consent-driven identity-assured API.<br>Presented unmodified to clinician in COPD dashboard.
Home NIV monitoring	Daily home NIV data available for clinician review in NHS GG&C Ehealth IG/security approved ResMed AirView remote-monitoring platform.	Daily home NIV data acquired via consent-driving identity assured API from AirView remote-monitoring platform.<br>Presented unmodified to clinician in COPD dashboard.
COPD self-management, generic	Paper or digital information (British Lung Foundation, or MyLungsMyLife Scottish website developed by respiratory MCN) supports clinical explanations, including 'traffic light' system for recognising and managing exacerbations.	RECEIVER patient dashboard and linked COPD support website contains content matching paper and digital information currently provided.
COPD self-management, individualised	Clinician documents for patient on paper (then in letter or other area of electronic health record) antibiotic and/or prednisolone dose	Clinician documents in RECEIVER dashboard antibiotic and/or prednisolone dose for exacerbation. This is visible at

	for exacerbation. Prescription provided.	self-management section of patient dashboard, and exported to GP and GG&C electronic health record. Prescription provided.
Patient unscheduled contact with COPD clinical team	Patient provided with leaflet and business card with answerphone/email for relevant clinical teams (hospital CNS, community resp team, clinical physiology team). Standard written text and email auto-reply on clinician availability and signposting for emergencies provided. No admin support, ad-hoc approach to managing messages by clinical team.	Patient can submit message via patient dashboard. Standard advice – identical to current clinical care – on clinician availability and signposting for emergencies provided on screen. Email and text message alerts patient to any new message from clinical team. Clinician messaging dashboard highlights new and unresolved messages. RECEIVER project manager oversees and ensures appropriate response to patient, and any escalation.
Scheduled patient reviews	Appointments made via Trakcare or telephone/paper diary by clinical team. Email and text message reminder alerts sent. Telephone calls, text or email queries, videocalls (NHS attend anywhere) used in place of hospital or domiciliary attendance, when possible.	Current routine-clinical care supplemented by clinician-patient messaging via RECEIVER dashboards. E.g. messaging used for appointment scheduling, information gathering to add value-efficiency to telephone or face-face consultation.
Clinical documentation	Paper notes (scanned to EHR), GG&C clinical portal clinical notes (in EHR), dictated-transcribed letters. COPD shared documentation e-form in clinical portal (previously trakcare) q3-4 2019. Documentation siloed: often not visible/shared across primary-secondary care split.	Structured documentation and free text clinical notes in clinician RECEIVER dashboard. Content matches COPD shared documentation clinical portal e-form. Clinical summary, clinical notes and anticipatory care plan exported as pdf from dashboard to EHR (SCI store and clinical portal), visible to all primary and secondary care team.
Recording – recognition of clinical episodes / events	Trakcare episodes and clinician-dependant recognition and narrative documentation in EHR notes or letters.	Trakcare episodes linked, and clinician recognition supported by symptom diary and messaging system. Episodes-events logged unmodified in timeline in RECEIVER clinician dashboard.
Patient data storage and management	NHS GG&C Ehealth systems.	NHS GG&C Ehealth systems.
