# Supplementary material for: Remote-Management of COPD: Evaluating the Implementation of Digital Innovation to Enable Routine Care (RECEIVER): the protocol for a feasibility and service adoption observational cohort study
Source: BMJ Open Respir Res. 2021 Aug 30;8(1):e000905. doi: 10.1136/bmjresp-2021-000905 (PMC8407208; doi:10.1136/bmjresp-2021-000905)
Supplement: Supplementary data [file bmjresp-2021-000905supp003.pdf]

Supplementary material 3: RECEIVER Trial – Data Storage in NHS GG&C Azure tenancy

The working components of the COPD digital service are maintained within the NHS GG&C Lenus account. The databases containing the patient data from the Receiver trial, and the historical and contemporary control cohort data from NHS GG&C SafeHaven is maintained in a separate account, with restricted access. Planned analyses of these datasets is subject to LPAC approvals, and SafeHaven SOPs to ensure only de-identified data is shared.

DYNAMIC Connectivity and Data Flows

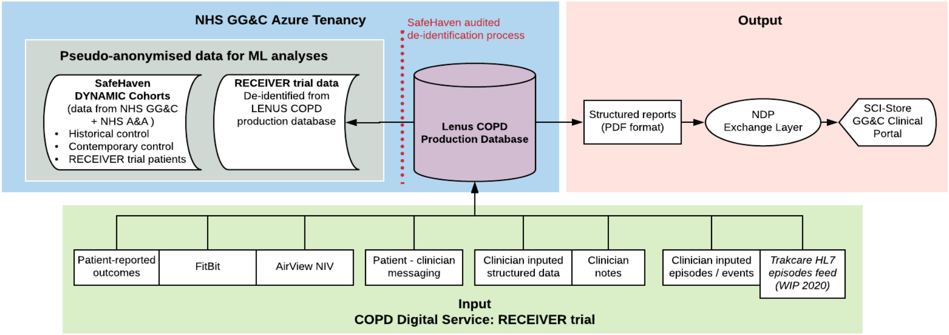

StormID - NHS GG&C Data Processing Agreement
Schedule 1 = Access to Lenus COPD Production Database and associated architecture for technical support and dataset preparation
Schedule 2 = Access to pseudo-anonymised data for ML analyses

COPD cohort inclusion
SMR 1 or SMR 4 ICD COPD J44 or J43 emphysema (not J430)
or LES diagnosis COPD
Age >40

Historical 1st Jan 2010 - 31st Dec 2016

Contemporary 1st Sept 2017 - 31st Aug 2020
not enrolled/screened for RECEIVER trial

Linked Patients enrolled in RECEIVER trial, match studyID to SafeHaven ID
