# Supplementary material for: Remote-Management of COPD: Evaluating the Implementation of Digital Innovation to Enable Routine Care (RECEIVER): the protocol for a feasibility and service adoption observational cohort study
Source: BMJ Open Respir Res. 2021 Aug 30;8(1):e000905. doi: 10.1136/bmjresp-2021-000905 (PMC8407208; doi:10.1136/bmjresp-2021-000905)
Supplement: Supplementary data [file bmjresp-2021-000905supp001.pdf]

Supplementary material 1: RECEIVER COPD Patient-web portal questionnaires

COPD Patient App v5.0

Patient Reported Outcome Flows

Daily

Symptom diary

1. How are you feeling today?
(1) Better than usual
(2) Normal/usual
(3) Worse than usual
(4) Much worse than usual

2. How is your breathing today?
(1) Better than usual
(2) Normal/usual
(3) Worse than usual
(4) Much worse than usual

3. Do you have a cold or flu today?
   - Yes
   - No

CAT (score /40)

4. (0) I never cough
(1)
(2)
(3)
(4)
(5) I cough all the time

5. (0) I have no phlegm (mucus) in my chest at all
(1)
(2)
(3)
(4)
(5) My chest is completely full of phlegm (mucus)

Symptom diary additional questions

How difficult is it to bring up phlegm when you cough?
(1) Not difficult
(2) A little difficult
(3) Quite difficult
(4) Very difficult

What consistency is your phlegm?
(1) Watery
(2) Sticky liquid
(3) Semi-solid
(4) Solid

What colour is your phlegm?
(1) White
(2) Yellow
(3) Green
(4) Dark green

6. (0) My chest does not feel tight at all
(1)
(2)
(3)
(4)
(5) My chest feels very tight

7. (0) When I walk up a hill or one flight of stairs I am not breathless
(1)
(2)
(3)
(4)
(5) When I walk up a hill or one flight of stairs I am very breathless

8. (0) I am not limited doing any activities at home
(1)
(2)
(3)
(4)
(5) I am very limited doing activities at home

9. (0) I am confident leaving my home despite my lung condition
(1)
(2)
(3)
(4)
(5) I am not at all confident leaving my home because of my lung condition

10. (0) I sleep soundly
(1)
(2)
(3)
(4)
(5) I don't sleep soundly because of my lung condition

11. (0) I have lots of energy
(1)
(2)
(3)
(4)
(5) I have no energy at all

Weekly

Symptom diary

1. How are you feeling today?

(1) Better than usual
(2) Normal/usual
(3) Worse than usual
(4) Much worse than usual

2. How is your breathing today?

(1) Better than usual
(2) Normal/usual
(3) Worse than usual
(4) Much worse than usual

3. Do you have a cold or flu today?

- Yes
- No

4. Have you increased your usual breathing treatment this week? (e.g. inhalers, nebulisers, tablets)

- Yes
- No

5. Have you taken antibiotics this week?

- Yes
- No

6. Have you visited your GP this week?

- Yes
- No

7. Have you visited your hospital doctor this week?

- Yes
- No

CAT (score /40) (For each of the following questions, please select the number that best describes you currently.)

8. (0) I never cough

(1)
(2)
(3)
(4)
(5) I cough all the time

9. (0) I have no phlegm (mucus) in my chest at all

(1)
(2)
(3)
(4)
(5) My chest is completely full of phlegm (mucus)

Symptom diary additional questions

How difficult is it to bring up phlegm when you cough?

- Not difficult
- A little difficult
- Quite difficult
- Very difficult

What consistency is your phlegm?

- Watery
- Sticky liquid
- Semi-solid
- Solid

What colour is your phlegm?

- White
- Yellow
- Green
- Dark green

10. (0) My chest does not feel tight at all
(1)
(2)
(3)
(4)
(5) My chest feels very tight

11. (0) When I walk up a hill or one flight of stairs I am not breathless
(1)
(2)
(3)
(4)
(5) When I walk up a hill or one flight of stairs I am very breathless

12. (0) I am not limited doing any activities at home
(1)
(2)
(3)
(4)
(5) I am very limited doing activities at home

13. (0) I am confident leaving my home despite my lung condition
(1)
(2)
(3)
(4)
(5) I am not at all confident leaving my home because of my lung condition

14. (0) I sleep soundly
(1)
(2)
(3)
(4)
(5) I don’t sleep soundly because of my lung condition

15. (0) I have lots of energy
(1)
(2)
(3)
(4)
(5) I have no energy at all

MRC (score /4)

Please tick in the box that applies to you (one box only):

1. I only get breathless with strenuous exercise
1. I get short of breath when hurrying on the level or walking up and slight hill
2. I walk slower than people of the same age on the level because of breathlessness or have to stop for breath when walking at my own pace on the level
3. I stop for breath after walking about 100 yards or after a few minutes on the level
4. I am too breathless to leave the house or I am breathless when dressing

Every 4th week

Symptom diary

16. How are you feeling today?
(1) Better than usual
(2) Normal/usual
(3) Worse than usual
(4) Much worse than usual

17. How is your breathing today?
(1) Better than usual
(2) Normal/usual
(3) Worse than usual
(4) Much worse than usual

18. Do you have a cold or flu today?
- Yes
- No

19. Have you increased your usual breathing treatment this week? (e.g. inhalers, nebulisers, tablets)
- Yes
- No

20. "Have you taken a rescue pack or an acute course of antibiotics or steroids prescribed by a doctor for your COPD this week? *This does not include long-term antibiotics."?*
- Yes
- No

21. Have you visited your GP this week?
- Yes
- No

22. Have you visited your hospital doctor this week?
- Yes
- No

CAT (score /40) (For each of the following questions, please select the number that best describes you currently.)

23. (0) I never cough
(1)
(2)
(3)
(4)
(5) I cough all the time

24. (0) I have no phlegm (mucus) in my chest at all
(1)
(2)
(3)
(4)

(5) My chest is completely full of phlegm (mucus)

Symptom diary additional questions

How difficult is it to bring up phlegm when you cough?

- Not difficult
- A little difficult
- Quite difficult
- Very difficult

What consistency is your phlegm?

- Watery
- Sticky liquid
- Semi-solid
- Solid

What colour is your phlegm?

- White
- Yellow
- Green
- Dark green

25. (0) My chest does not feel tight at all
(1)
(2)
(3)
(4)
(5) My chest feels very tight

26. (0) When I walk up a hill or one flight of stairs I am not breathless
(1)
(2)
(3)
(4)
(5) When I walk up a hill or one flight of stairs I am very breathless

27. (0) I am not limited doing any activities at home
(1)
(2)
(3)
(4)
(5) I am very limited doing activities at home

28. (0) I am confident leaving my home despite my lung condition
(1)
(2)
(3)
(4)
(5) I am not at all confident leaving my home because of my lung condition

29. (0) I sleep soundly
(1)
(2)

(3)
(4)
(5) I don't sleep soundly because of my lung condition

30. (0) I have lots of energy
(1)
(2)
(3)
(4)
(5) I have no energy at all

MRC (score /4)

Please tick in the box that applies to you (one box only):

1. I only get breathless with strenuous exercise
1. I get short of breath when hurrying on the level or walking up and slight hill
2. I walk slower than people of the same age on the level because of breathlessness or have to stop for breath when walking at my own pace on the level
3. I stop for breath after walking about 100 yards or after a few minutes on the level
4. I am too breathless to leave the house or I am breathless when dressing

Every month we are asking some additional research questions to help us understand the impact of COPD on your quality of life. These questions will take a further couple of minutes, but can be skipped if you prefer *(with opt in/out click option)*

Quality of Life (EQ5D)

Mobility

- I have no problems in walking about
- I have slight problems in walking about
- I have moderate problems in walking about
- I have severe problems in walking about
- I am unable to walk about

Self-care

- I have no problems washing or dressing myself
- I have slight problems washing or dressing myself
- I have moderate problems washing or dressing myself
- I have severe problems washing or dressing myself
- I am unable to wash or dress myself

Usual activities (e.g. work, study, housework, family or leisure activities)

- I have no problems doing my usual activities
- I have slight problems doing my usual activities
- I have moderate problems doing my usual activities
- I have severe problems doing my usual activities
- I am unable to do my usual activities.

Pain/discomfort

- I have no pain or discomfort
- I have slight pain or discomfort
- I have moderate pain or discomfort

- I have severe pain or discomfort
- I have extreme pain or discomfort

Anxiety/depression

- I am not anxious or depressed
- I am slightly anxious or depressed
- I am moderately anxious or depressed
- I am severely anxious or depressed
- I am extremely anxious or depressed

- We would like to know how good or bad your health is TODAY.
- This scale is numbered from 0 to 100.
- 100 means the best health you can imagine.
  0 means the worst health you can imagine.
- Mark an X on the scale to indicate how your health is TODAY.
- Now, please write the number you marked on the scale in the box below.

YOUR HEALTH TODAY = ☐

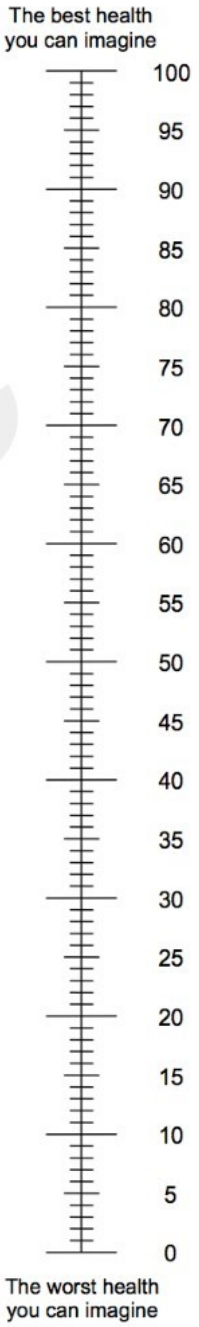

Sample
